# Statistical mechanics of soft collagenous tissues: a constitutive model with damage for cerebral bridging veins

**DOI:** 10.1007/s00249-026-01839-2

**Published:** 2026-04-16

**Authors:** Silvia García-Vilana, David Sánchez-Molina, Hamed Abdi, Vafa Rahimi-Movaghar

**Affiliations:** 1 Dept. Strength of Mat & Eng Struct (GAECEQS-EPSEVG), UPC-BarcelonaTech, Vilanova i la Geltrú, Spain; 2 Sina Trauma and Surgery Research Center, TUMS, Tehran, Iran; 3 Dept. Mech. Eng. (TECNOFAB-EEBE), UPC-Barcelona Tech, Barcelona, Spain

**Keywords:** Constitutive model with damage, Parasagittal bridging veins, Tissue microstructure, Collagenous tissue, Soft tissue, Statistical mechanics.

## Abstract

A novel constitutive material model for the collagenous tissue of bridging veins is presented in this article. The walls of blood vessels are structurally reinforced by the collagen fibers, which are interconnected with an elastin matrix. Collagen fibers are oriented according to a specific probability distribution, with a greater abundance of fibers along the longitudinal axis. It is possible to analyze the collective mechanical behavior of collagen fibers using statistical mechanics techniques. The article describes how to derive the macroscopic behavior of collagenous tissue using the formalism of the canonical ensemble of statistical mechanics. One of the innovations of this formulation is the incorporation of a damage variable that increases significantly under excessive stretching. This formalism enables the derivation of a constitutive material model that is thermodynamically consistent, in which entropy increases irreversibly with the level of damage. In the model, an increase in damage results in a permanent increase in entropy. Furthermore, for a given level of damage, an increase in strain induces organization in the fibers, which is consistent with the behavior of an entropically elastic material. In addition, the thermodynamic properties of the model account for specific empirical observations, such as the slight contraction that occurs in a blood vessel when it is heated. The model’s mechanical suitability was assessed by subjecting it to uniaxial tensile tests on bridging veins. The findings provide strong evidence that the proposed constitutive model accurately reproduces the stress–strain curves ($R^2_{adj}> 0.99$).

## Introduction

Constitutive models for collagenous tissues in the walls of blood vessels are of great practical interest because of the importance of computational biomechanics, where such models are required (Migueis et al. 2019; Abdi et al. 2023; Fernandes and Silveira 2024; Gomes et al. 2024). For instance, it is widely recognized that an excessive stress or strain in certain blood vessels, such as the parasagittal cerebral bridging veins (BVs), often leads to rupture and subsequent bleeding (Depreitere et al. 2006; Monson et al. 2019; Abdi et al. 2025). In fact, BVs play a crucial role in draining blood from the brain, and their rupture is a key factor in the onset of acute subdural hematoma (ASDH) (Costa et al. 2020; Miller and Nader 2014; Ye et al. 2024). Worldwide, the incidence of ASDH suggests that road crashes cause some 16 million cases of subdural hematoma, with 1.25 million being severe cases (García-Vilana et al. 2021). On the basis of these numbers, it is possible to conclude that ASDH is a significant issue in road traffic collisions and other traumatic situations; as a result, a great deal of research has been devoted to the mechanical constitutive modeling of BVs (Vito and Dixon 2003; Fernandes and Silveira 2024; Monea et al. 2014; Famaey et al. 2015; García-Vilana et al. 2021).

Few studies have explored the thermodynamic basis of injury generation in impact trauma, where damage is modeled as a dissipative mechanical process using continuum damage mechanics (CDM) grounded in irreversible thermodynamics (Neal-Sturgess 2002). Similarly, statistical mechanics applied to the microcomponents of biological tissues has proven useful for deriving constitutive models of mechanical behavior under irreversible damage by considering microstructure (García-Vilana and Sánchez-Molina 2025).

The hierarchical structure of biological tissues has also been studied to model mechanical behavior both within the framework of CDM (Balzani et al. 2012; Blanco et al. 2015; Li 2016; Rausch et al. 2017) and from the perspective of mechanobiology (Doblaré 2015; Sánchez-Molina and García-Vilana 2024; Sáez et al. 2012). Several of the authors involved in these studies have also addressed the issue of thermodynamic consistency (Sáez et al. 2012; Klika et al. 2014; Popović and Minceva 2020). In fact, statistical mechanics offers a powerful framework that simultaneously accounts for both microstructural organization and thermodynamic principles.

This study addresses two key aspects of the mechanical and thermodynamic behavior of BVs:

1. On the one hand, like many collagenous soft tissues, BVs exhibit a convex stress–strain curve, as illustrated in Fig. 4. This feature implies that the slope of the stress–strain curve increases significantly with strain. In the literature, this feature is frequently modeled by means of “exponential-type” models. For the uniaxial case, this results in an exponential constitutive relationship of the form $\sigma (\varepsilon ) \propto \exp (a \varepsilon ^2)$ (*σ*: stress; *\varepsilon*: strain; and *a> 0* a real constant). The Fung–Deng model (Deng et al. 1994; Yang et al. 2006), the Holzapfel–Kroon model (Holzapfel et al. 2000; Kroon and Holzapfel 2008), and the Natali–Gregersen model (Natali et al. 2009), among others, are examples of models that use exponential-type relationships (Sanchez-Molina et al. 2014; Ho and Kleiven 2007). These models vary in complexity; however, they are all generalizations of the classical hyper-elastic model proposed by Fung (2013). Although exponential-type models adequately fit the data, there is a scarcity of deep theoretical explanations for them. This paper offers one from the field of statistical mechanics.
2. On the other hand, there is a significant difference between the elasticity of rubber (and soft collagenous tissues) and that of hard deformable solids with respect to the thermodynamic characteristics. It turns out that in hard solids such as steel, concrete, or wood, the deformation does not produce significant changes in entropy in the range of reversible deformations, while in a material such as rubber or amorphous polymers formed by chemically cross-linked chains, where stretching induces a decrease in entropy due to chain alignment and the associated cooling effect (Treloar 1976; Flory 1666). The distinctions in deformation mechanisms between soft materials, including rubber, and hard materials have prompted the deduction that the former demonstrate *entropic elasticity*, whereas the latter exhibit *energetic elasticity* (Guth 1966; Kloczkowski et al. 1991).
3. Even J.P. Joule (1859) made an early observation that when vulcanized rubber was subjected to a tensile force, it underwent a transient cooling effect at low strain levels before transitioning to a heating effect as strain increased (Joule 1859). This rubber-like phenomenon is observed in numerous soft tissues of the human body. For instance, it is well understood that rubber undergoes a contraction when its temperature increases while subjected to tensile conditions. Moreover, while C.S. Roy (1880−82) first observed this phenomenon many years ago, it is still relatively unknown that heating causes the contraction of arteries in both humans and animals (Roy 1881).

The statistical mechanics applied to collagen fibers in soft tissues provides an explanation for the “exponential-type” constitutive relations. The collagen fibers are straightened and ordered through stretching. This reduces their entropy and enables us to account for the stress-strain relationships observed in collagenous soft tissues. This article derives a constitutive equation characteristic of entropic elasticity that adequately explains the mechanical behavior of BVs using the methods of statistical mechanics.

## Materials and methods

The materials used to check the adequacy of the proposed constitutive model consisted of fresh BV specimens harvested from forensic autopsies at the Legal Medicine and Forensic Science Institute of Catalonia (IMLCFC). All the specimens were initially removed for a complementary medical-legal investigation. IMLCFC’s Research and Ethics Committee granted approval for this investigation. Twelve sections of the meningeal-cortex space (including the sub-arachnoid space, the upper portion of the cerebral cortex, and the meninges) were extracted from autopsies of *post mortem* human subjects (PMHSs) for the mechanical tests. No specimen was obtained from any PMHS that had been previously diagnosed with cerebral vessel pathology. After being received, the sections were stored in airtight containers that preserved their natural degree of hydration and were refrigerated for a maximum of 96 hours. Each BV was meticulously dissected from the meninges and surrounding brain tissue, obtaining *n = 23* BV specimens, from *N = 12* different donors. The age range spans 50 to 92 years (mean ± SD = *70.7 ± 11.6* years). Immediately thereafter, a tensile test was performed on each BV specimen, stretching it until its complete rupture.

### Statistical mechanics of collagenous tissues

Statistical mechanics was used in this research in order to formulate the BV constitutive model. The purpose of statistical mechanics is to describe the behavior of a macroscopic system composed of elementary constituents, based on the energy exchange and the mutual influence they exert on each other. In our case, the collection of collagen fibers that constitute the walls of BVs interact and function as the elementary constituents of the model. These collagen fibers collectively form the structural reinforcement of the walls of these blood vessels (Pukaluk et al. 2023). Thus, within the framework of a statistical mechanics, the collection of collagen fibers constitutes a thermodynamic system, i.e., a canonical ensemble in interaction.

The *canonical ensemble formalism* of statistical mechanics generates a *partition function* $Z(\varepsilon ,\delta ;\beta )$, also called *Zustandssumme* (‘sum over states’) (Dean et al. 2005), which will be computed in Sect. "The partition function *Z* of the model". The function *Z* depends on the macroscopic magnitudes $(\varepsilon ,\delta ;\beta )$, where *\varepsilon* is the local longitudinal strain and *δ* the local damage of the BV and the parameter *β* characterizes the change in entropy *S* as the internal energy *U* of the system increases (Swendsen 2017):

1 $$\begin{aligned} \beta = \left( \frac{\partial S}{\partial U}\right) _{\varepsilon ,\delta } \end{aligned}$$

For many hydrostatic systems, the quenched-disorder parameter satisfies *β> 0*, but in the case of collagenous tissues, where a progressive straightening of collagen fibers diminishes disorder and waviness of collagen fibers as strain increases, *β < 0* in systems that become more ordered as energy increases. This behavior arises because, at a fixed strain level, the admissible energy states of each fiber are bounded, in contrast to the unbounded nature of molecular kinetic energy in a hydrostatic system. Furthermore, as discussed above, the parameter *β* is not directly associated with the ordinary thermal temperature; instead, it plays the role of a quenched disorder parameter in the present framework, which is reminiscent of what happens in systems of *negative temperature* (Ramsey 1956; Baldovin et al. 2021).

The thermodynamic potentials and the stress-strain relationship can be derived from the *Z* function. For instance, *Z* allows us to obtain the free Helmholtz energy density *Ψ*:

2 $$\begin{aligned} \Psi (\varepsilon ,\delta ;\beta ) = -\frac{\ln Z(\varepsilon ,\delta ;\beta )}{\beta } \end{aligned}$$

This function *Ψ* is also called strain-damage energy density function (SDEDF), and from it, all the necessary constitutive relations can be derived. Thus, the stress *σ* is given by:

3 $$\begin{aligned} \sigma (\varepsilon ,\delta ;\beta ) = \frac{\partial \Psi (\varepsilon ,\delta ;\beta )}{\partial \varepsilon } \end{aligned}$$

Moreover, the internal energy (*U*) and entropy (*S*) can be determined by employing the conventional Maxwell–Boltzmann formalism:

4 $$\begin{aligned} U(\varepsilon ,\delta ;\beta ) = -\frac{\partial \ln Z}{\partial \beta }, \qquad S(\varepsilon ,\delta ;\beta ) = \beta ^2 \frac{\partial }{\partial \beta }\left( \frac{\ln Z}{\beta } \right) \end{aligned}$$

### The partition function *Z* of the model

In order to calculate $Z(\varepsilon ,\delta ;\beta )$, we introduce the notions of macrostate and microstate. In a thermodynamic system, several macroscopic magnitudes characterize a system’s macrostate (in our case, the strain and the amount of damage) (Toussaint and Pride 2002; Shenker 2017). Each macrostate is compatible with many microstates or configurations of the microelements forming the system (in our case, the configuration of collagen fibers). Based on the number of microstates compatible with each macrostate, Statistical Mechanics allows us to determine the most probable macrostate, which is proportional to the number of compatible microstates, and the average behavior for each macrostate (García-Vilana and Sánchez-Molina 2025; Wallace 2020). For practical purposes, it is more useful to compute the partition function by summing over the energies of the macrostates than over the microstates; to do this, it is sufficient to count the number of microstates with a given energy. Since the differences between different energy states are very small, the sum can be replaced by an integral.

**Fig. 1:**
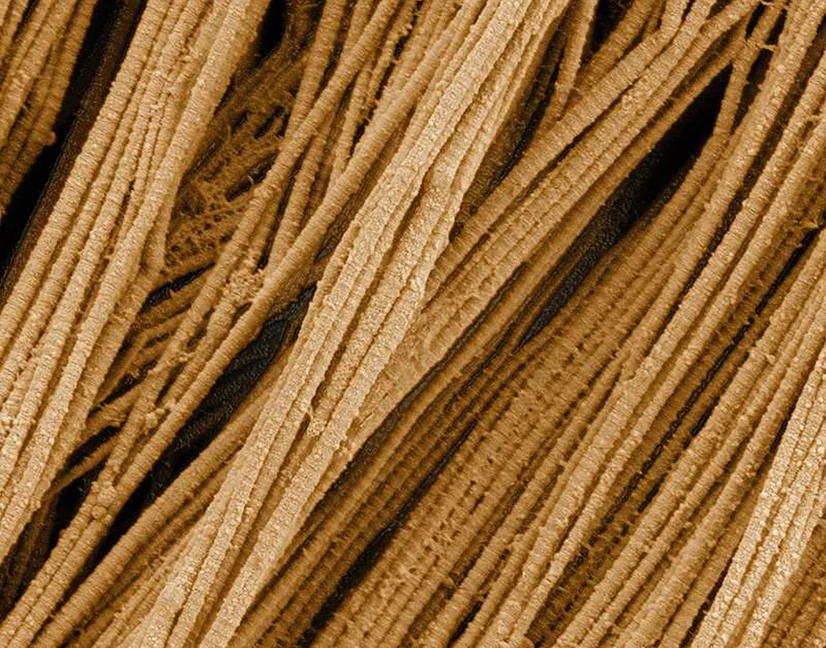
Scanning electron microscopy of collagen fibers (CC, Credit: Thomas J. Deerinck and Mark H. Ellisman, NCMIR, University of Californa San Diego)

Furthermore, in the proposed model it is assumed that the energy in terms of the state variables $(\varepsilon ,\delta )$ has the form:

5 $$\begin{aligned} E(\varepsilon ,\delta ) = Y(1-\delta )\varepsilon ^2/2 + o(\varepsilon ^3) \ge 0 \end{aligned}$$

where *Y* is the total stiffness associated with the collagen fibers, $0 \le \delta \le 1$ is the damage variable and *\varepsilon* is the local strain of the collagenous tissue. This choice is based on the limiting behavior of an elastic material with low strain damage. With these considerations, the partition function can now be written as:

6 $$\begin{aligned} Z(\varepsilon ,\delta ;\beta ) = g_0 e^{-\beta E(0,\delta )} + \int _0^{\varepsilon } g(\bar{\varepsilon }) e^{-\beta E(\bar{\varepsilon },\delta )}\ \text {d}\bar{\varepsilon } \end{aligned}$$

where $g_0$ is the number of fibers initially aligned with the tensile force direction and the function *g(\varepsilon )* accounts for the number of microstates compatible with a macrostate with strain *\varepsilon*. This number depends on the random distribution of collagen fiber lengths (see Fig. 1).

Since *β < 0*, as established in Eq. (1) and discussed in Sect. "Statistical mechanics of collagenous tissues", the function $\beta E(\varepsilon ,\delta )$ is monotonic increasing. Therefore, by the second mean value theorem for integrals (Wituła et al. 2012), applied to the monotonic increasing function $f(\bar{\varepsilon }):= g(\bar{\varepsilon }) e^{-\beta E(\bar{\varepsilon },\delta )}$, there exists a real number *0< θ < 1* such that:

7 $$\begin{aligned} Z(\varepsilon ,\delta ;\beta ) = g_0 \underbrace{e^{-\beta E(0,\delta )}}_{=1} + e^{-\beta E(\varepsilon ,\delta )} \underbrace{\int _{\theta \varepsilon }^{\varepsilon } g(\bar{\varepsilon }) \ \text {d}\bar{\varepsilon }}_{g_1} = g_0 + g_1 e^{-\beta E(\varepsilon ,\delta )} \end{aligned}$$

we will see that the ratio $g_1/g_0> 0$ of the two numbers can be found empirically without the need to know precisely the details of the function *g(\varepsilon )* or the number *θ*.

### Microdamage evolution and stress-strain relationship

To complete the model, it is necessary to specify how microdamages evolve within the BV wall (Lemaitre 1985). In the proposed model, the probability of fiber failure is assumed to follow a Weibull distribution with parameters $(\varepsilon _d,n)$. Therefore, the proportion of collagen fibers exhibiting microdamage at time *t* in an arbitrary loading process would be $\delta _t$:

8 $$\begin{aligned} \delta _t = \max _{t \ge 0}\,\{1 - e^{-(\varepsilon _t/\varepsilon _d)^n}\} \end{aligned}$$

where $\epsilon _t$ is the longitudinal strain along the BV in time *t*. This equation implies that the expected number of collagen fibers harmed would depend on the highest strain $\varepsilon _{\max } = \max _{t\ge 0} \varepsilon _t$ reached during the loading process. The specific choice of this law is grounded in stochastic failure models and in extreme value theory, since both stiffness and strength are typically governed by the weakest fibers or by the most severe local stress concentrations (Sánchez-Molina et al. 2015; Li 2016). An equivalent expression for microdamage evolution in terms of time derivatives $\dot{\delta }_t$ and $\dot{\varepsilon }_t$ is:

9 $$\begin{aligned} \dot{\delta }_t = {\left\{ \begin{array}{ll} n(\varepsilon _t^{n-1}/\varepsilon _d^n)\exp (-\varepsilon _t^n/\varepsilon _d^n)\dot{\varepsilon }_t, & \varepsilon _t \ge \max _{0 \le \tau \le t}\varepsilon _{\tau }\\ 0, & \varepsilon _t < \max _{0 \le \tau \le t}\varepsilon _{\tau } \end{array}\right. } \end{aligned}$$

This evolution law is consistent with the second law of thermodynamics. It is worth noting that this principle, in conjunction with the model-derived Eq. (11), implies that *δ* cannot decrease; consequently, the time derivative of the damage is $\dot{\delta }_t \ge 0$. Finally, the stress can be calculated by introducing the partition function of (7) in Eq. (2), then using (3), it results in:

10 $$\begin{aligned} \sigma (\varepsilon ,\delta ;\beta ) = \frac{k}{k+e^{\beta Y (1-\delta )\varepsilon ^2}}Y(1-\delta )\varepsilon \end{aligned}$$

where $$k = (g_1/g_0)$$.

### Experimental setting

**Fig. 2:**
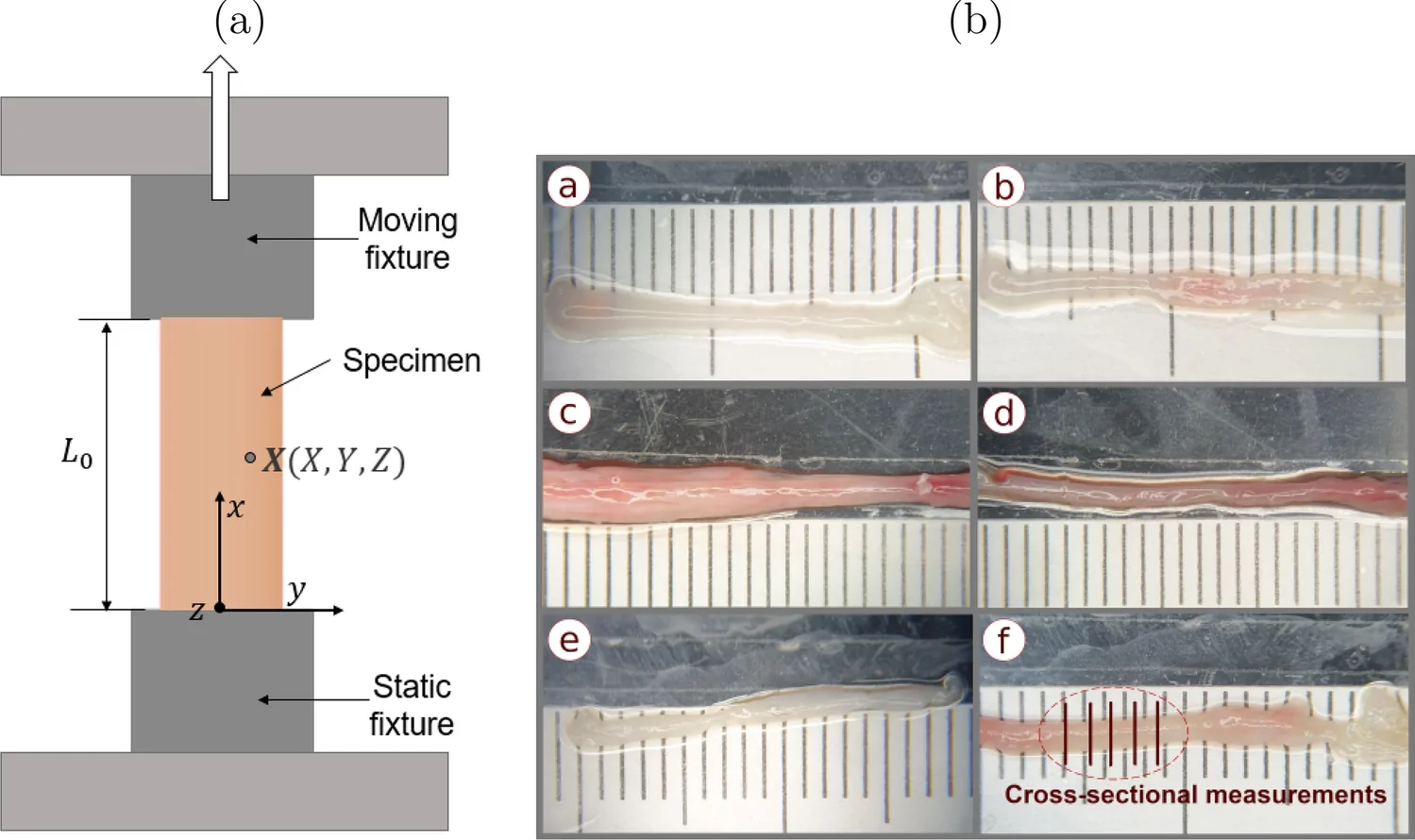
(**a**) Schematic of the test setting, the lower fixture is at rest and the upper fixture is movable, (**b**) Pre-test photographs of six specimens of BVs, in (f) an example of the multiple measurement to obtain outer diameter average

In order to assess the adequacy of the new constitutive model, the current study repurposes the experimental data acquired during a preliminary investigation (Sánchez-Molina et al. 2021). In this prior work *n=23* BV specimens were subjected to uniaxial tensile testing (see Fig. 2a). Each BV was photographed using a camera coupled to a stereo microscope SMZ-168, both from Motic^®^ (see Fig. 2b).

**Fig. 3:**
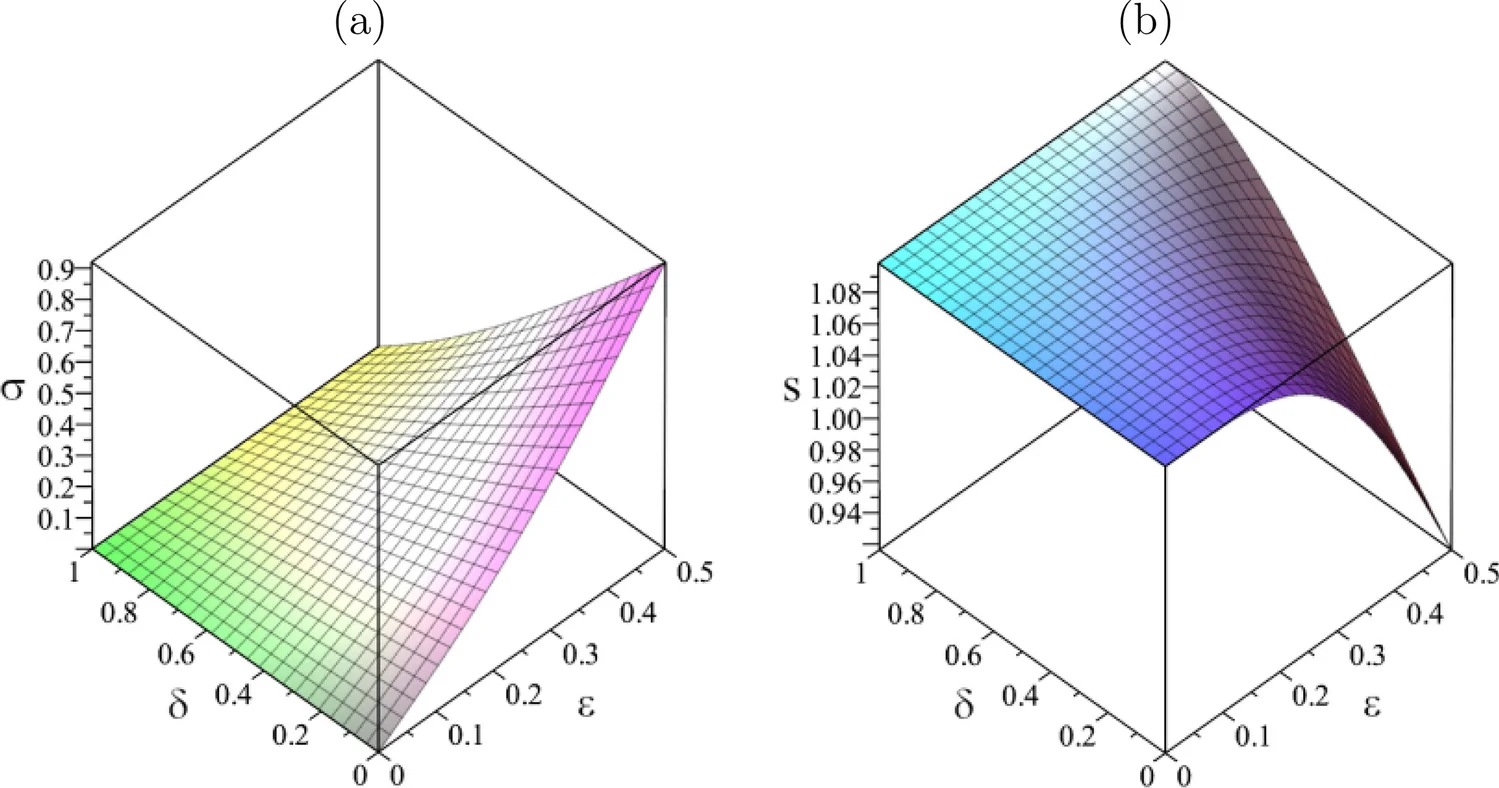
(**a**) Stress (*σ*) with respect to strain (*\varepsilon*) and damage (*δ*), (**b**) Entropy (*S*) with respect to *\varepsilon* and *δ*

The applied load was measured with a 20 N load cell HBM^®^, as described in prior empirical study (Sánchez-Molina et al. 2021). Tensile tests were conducted using a UTM Zwick-Roell^®^ (model Allround-Table-Top^®^). The strain rates observed during uniaxial tensile tests varied between 0.001 and 1.20 s$^{-1}$. The moderate strain rates resulted in negligible viscoelastic effects, as quantified in (Famaey et al. 2015; García-Vilana et al. 2021; Lee and Haut 1989) for the same sample. For this reason, many authors place emphasis on elastic properties (Fernandes and Silveira 2024; Famaey et al. 2015). Many specimens in the sample exhibited a final elongation around 50% (see Fig. 3), the infinitesimal strain theory was deemed unsuitable; consequently, finite-strain measurements were implemented (Sánchez-Molina et al. 2021).

## Results

In Sect. "Consequences of the model" some noticeable consequences of the constitutive model associated with partition function of (7) are derived and discussed. Sect. "Stress-strain curves" is the critical part of the results showing how the model accurately represents the experimental data. The subsequent Sect. "Other thermodynamic issues" elaborates on the initial observations that heating induces vasoconstriction in blood vessels (Roy 1881), and provides an explanation based on the proposed model.

### Consequences of the model

The constitutive model detailed in Sects. "Stress-strain curves" and "Other thermodynamic issues" has the following properties: (a) for any value of the local damage variable *δ*, the stress-strain curve begins as a convex curve but transitions to a concave shape at a specific strain threshold. Immediately after reaching maximum stress, the amount of damage escalates to the extent that the slope of the curve becomes negative; and (b) the stiffness decreases with the damage: $(\partial \sigma /\partial \delta )_{\varepsilon } < 0$, for constant strain levels; these two properties are illustrated in Fig. 3a. This can be compared with other similar findings (Khajehsaeid et al. 2021). Furthermore, the following holds: (c) as in entropically elastic materials, the entropy (*S*) decreases with the strain for a constant level of damage: $(\partial S/\partial \varepsilon )_{\delta } < 0$, and (d) the entropy increases as the damage increases: $(\partial S/\partial \delta )_{\varepsilon }> 0$, as corresponds to an irreversible process (Amiri and Modarres 2014; Imanian and Modarres 2018). These other two aspects are illustrated in Fig. 3b.

### Stress-strain curves

**Fig. 4:**
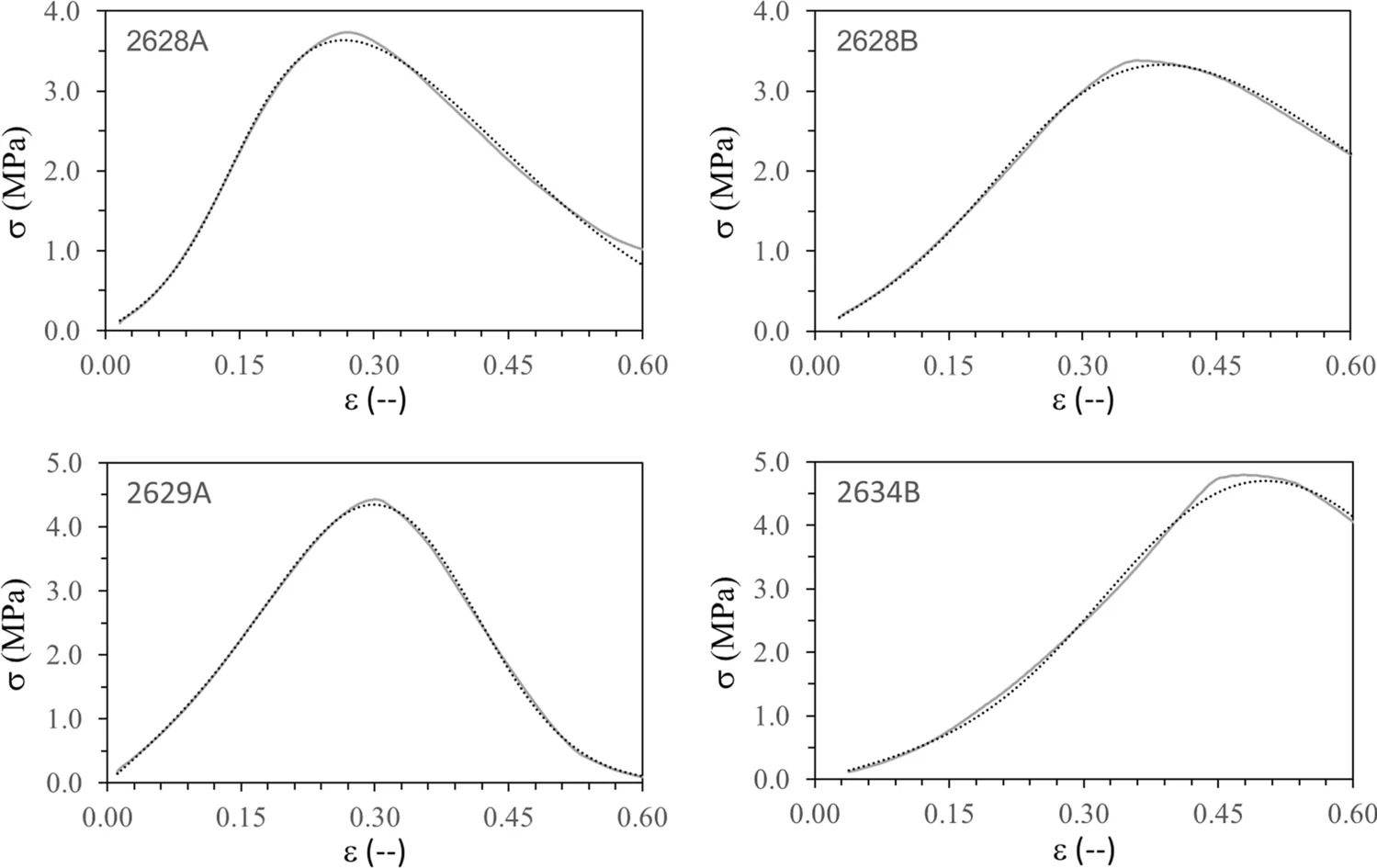
A comparison of predictions of the model (dotted lines) with experimental data (gray lines) for a set of four specimens (2628A, 2628B, 2629 A and 2634B)

The experiments conducted to assess the model’s adequacy comprised tensile tests that continued until failure. The stress-strain curves exhibited convex-concave-convex profile, as illustrated in Fig. 4. In all cases, a value of $R^2_{adj}>0.995$ was obtained, indicating that the model accurately represents the data.

The parameter values were as follows: the stiffness is *Y = 29.02± 3.65*, *k = 0.37± 0.12*, the quenched-disorder parameter $\beta = -2.92\pm 1.15$ [$10^{-3}$ m$^3$/kJ], the damage strain $\varepsilon _d = 0.44\pm 0.08$, and the damage exponent *n = 2.42± 0.55*. All the above material properties were estimated from specimens tested at strain rates within the interval $0.001 \le \dot{\varepsilon } \le 1.200\ \text {s}^{-1}$. For substantially higher strain rates, additional adjustments to the model may be required. The relationship with anthropometric variables, however, is not addressed in this research. It is well known that there is an age effect on the mechanical properties of BVs García-Vilana and (Sánchez-Molina 2022). A larger, gender- and age-balanced sample of BVs is necessary in order to understand the inherent variability in mechanical properties with regard to anthropometrical variables (Velázquez-Ameijide et al. 2021).

A limitation of our research is that it does not explain why autopsies often show that ASDH ruptures at the sagittal sinus-BV junction. Blood pressure, a stress rise, or local decrease in mechanical strength may explain the outcome. Further work is needed to determine whether any of these possibilities would explain those cases where the rupture does not occur along the BV.

### Other thermodynamic issues

As indicated in Sect. "Consequences of the model", entropy and damage are positively correlated. According to the second law of thermodynamics, the damage variable *δ* cannot decrease, since:

11 $$\begin{aligned} \left( {\frac{\partial S}{\partial \delta }}\right) _{\epsilon } = \underbrace{\frac{\beta ^2\varepsilon ^4}{4}}_{>0}\underbrace{(1-\delta )}_{>0} \underbrace{\frac{k e^{-\beta E(\varepsilon ,\delta )}}{\left( 1 +e^{-\beta E(\varepsilon ,\delta )}\right) ^2}}_{>0}> 0 \end{aligned}$$

This fact is incorporated into the Eq. (9) in the Sect. "Microdamage evolution and stress-strain relationship", by stipulating that the time derivative of damage is always $\dot{\delta }\ge 0$.

## Discussion

In TBI research, the proposed model provides a mechanobiological framework to numerically simulate the behavior of cerebral BVs in trauma situations, when there is a displacement of encephalon inside the cranium, a condition often implicated in the onset of acute subdural hematoma (ASDH). By characterizing damage evolution via a thermodynamically consistent formulation and modeling the entropic elasticity of collagen fibers, the model enhances the realism and predictive accuracy of finite element head models. Clinically, the model could be used as a complementary tool for evaluating vascular integrity in at-risk populations, such as elderly patients or individuals with cerebral atrophy. In forensic and criminalistic studies, the model allows simulations to be conducted to assess different hypotheses for trauma production. In computational biomechanics, this model provides a framework to simulate vascular damage progression under varied mechanical and thermal conditions. Its incorporation of entropy-based damage mechanics enables the reproduction of phenomena such as vasoconstriction under heating, which aligns with empirical observations. Future research may expand its applicability by integrating it with anatomically-detailed, subject-specific simulations of the cerebral vasculature.

In contrast to classical “exponential-type” constitutive models, which impose an *ad hoc* relationship of the form $\sigma (\varepsilon ) \propto \exp (a\varepsilon ^2)$, such as the Fung–Deng model (Deng et al. 1994), our framework derives exponential behavior naturally from the entropy-based alignment of collagen fibers under strain.

Structural anisotropy models like the Holzapfel–Gasser–Ogden (HGO) law (Holzapfel et al. 2000; Fehervary et al. 2020) account for fiber orientation and multilayer arterial mechanics but omit a thermodynamically consistent damage variable. Although the HGO formulation has been widely adapted to general soft tissues (Nolan et al. 2014), it lacks an explicit damage evolution grounded in irreversible thermodynamics.

On the other hand, CDM approaches introduce phenomenological internal variables to capture soft-tissue softening and residual strains (Balzani et al. 2012), yet they typically rely on empirical damage functions without microstructural derivation. In contrast, our model bases damage growth on a Weibull distribution tied to the maximum experienced strain, ensuring non-decreasing damage in accordance with the second law.

Reduced-order multilayer models for veins and arteries (Haider et al. 2024) capture pressure–diameter relations with numerous calibrated parameters, but assume fixed thermodynamic properties. By contrast, our five-parameter model compactly unifies entropic elasticity and irreversible damage, offering a robust platform for subject-specific computational simulations of vascular failure. Statistical-mechanics-based models have been applied to describe damage in cortical bone (García-Vilana and Sánchez-Molina 2025, 2024) where canonical ensembles are used; the present study extends this methodology to soft collagenous vessels.

We also note several general limitations of our study. First, the model is based on uniaxial mechanical tests. Although physiologically relevant loading is predominantly tensile —since these thin, slender tubular vessels are primarily stretched during relative motion between the brain and the skull, and other loading modes are unlikely to play a dominant mechanical role in vivo— this assumption should be empirically verified.

Second, available experimental evidence indicates that bridging veins exhibit weak or negligible strain-rate sensitivity within the moderate strain-rate range considered here (Famaey et al. 2015; García-Vilana et al. 2021). We acknowledge that under substantially different loading conditions —for example, at higher strain rates, during long-term creep, or under broadband oscillatory protocols— viscoelastic memory effects may become significant and would require a more comprehensive constitutive framework. However, such regimes fall outside the scope of the experiments and modeling objectives of the present study.

## Conclusion

A thermodynamically consistent constitutive model for the collagen fibers in the walls of cerebral bridging veins has been derived using the principles of statistical mechanics. The model accurately reproduces the observed stress–strain curves. In addition, a foundation of exponential laws in constitutive models is provided. Furthermore, the predictive capability of the model regarding the extent of microdamage is demonstrated to be positively correlated with the augmentation of entropy. Additionally, the model elucidates the reason behind the observed similarities in the behavior of entropically elastic bodies (rubber-like materials) and soft collagenous tissues.

## Abbreviations

ASDH: - Acute Subdural Hematoma
BV(s): - Bridging Vein(s)
CDM: - Continuous Damage Mechanics
DIC: - Digital Image Correlation
PC(A): - Principal Component (Analysis)
PMHS: - Post mortem Human Subject

## References

[CR1] Abdi H, Sanchez-Molina D, Garcia-Vilana S, Rahimi-Movaghar V (2023) Quantifying the effect of cerebral atrophy on head injury risk in elderly individuals: insights from computational biomechanics and experimental analysis of bridging veins. Injury 54(12):111125. 10.1016/j.injury.2023.111125 37867025

[CR2] Abdi H, Sanchez-Molina D, Garcia-Vilana S, Rahimi-Movaghar V (2025) Biomechanical perspectives on traumatic brain injury in the elderly: a comprehensive review. Prog Biomed Eng. 10.1088/2516-1091/ada654 39761631

[CR3] Amiri M, Modarres M (2014) An entropy-based damage characterization. Entropy 16(12):6434–6463. 10.3390/e16126434

[CR4] Baldovin M, Iubini S, Livi R, Vulpiani A (2021) Statistical mechanics of systems with negative temperature. Phys Rep 923:1–50. 10.1016/j.physrep.2021.03.007 31108672

[CR5] Balzani D, Brinkhues S, Holzapfel GA (2012) Constitutive framework for the modeling of damage in collagenous soft tissues with application to arterial walls. Comput Methods Appl Mech Eng 213:139–151. 10.1016/j.cma.2011.11.015

[CR6] Blanco S, Polindara CA, Goicolea JM (2015) A regularised continuum damage model based on the mesoscopic scale for soft tissue. Int J Solids Struct 58:20–33. 10.1016/j.ijsolstr.2014.12.013

[CR7] Costa JMC, Fernandes FAO, Alves de Sousa RJ (2020) Prediction of subdural haematoma based on a detailed numerical model of the cerebral bridging veins. J Mech Behav Biomed Mater 111:103976. 10.1016/j.jmbbm.2020.103976 32750673

[CR8] Dean DS, Lancaster D, Majumdar SN (2005) Statistical mechanics of combinatorial optimization problems with site disorder. Phys Rev E 72(2):026125. 10.1103/PhysRevE.72.026125 16196662

[CR9] Deng SX, Tomioka J, Debes JC, Fung YC (1994) New experiments on shear modulus of elasticity of arteries. Am J Physiol Heart Circ Physiol 266:H1–H10. 10.1152/ajpheart.1994.266.1.H1 8304490

[CR10] Doblaré M, Merodio J (2015) An introduction to biomechanics and mechanobiology. Biomechanics. Encyclopedia of Life Support Systems (EOLSS) Publications, 1-37

[CR11] Depreitere B, Van Lierde C, Vander Sloten J, Van Audekercke R, Van Der Perre G, Plets C, Goffin J (2006) Mechanics of acute subdural hematomas resulting from bridging vein rupture. J Neurosurg 104(6):950–9. 10.3171/jns.2006.104.6.950 16776340

[CR12] Famaey N, Cui ZY, Musigazi GU, Ivens J, Depreitere B, Verbeken E, Sloten JV (2015) Structural and mechanical characterisation of bridging veins: a review. J Mech Behav Biomed Mater 41:222–240. 10.1016/j.jmbbm.2014.06.009 25052244

[CR13] Fehervary H, Maes L, Vastmans J, Kloosterman G, Famaey N (2020) How to implement user-defined fiber-reinforced hyperelastic materials in finite element software. J Mech Behav Biomed Mater 110:103737. 10.1016/j.jmbbm.2020.103737 32771879

[CR14] Fernandes FAO, Silveira CIC (2024) The significance of cross-sectional shape accuracy and non-linear elasticity on the numerical modelling of cerebral veins under tensile loading. Biology 13:16. 10.3390/biology13010016 PMC10813171 38248447

[CR15] Flory PJ (1976) Statistical thermodynamics of random networks. Proceedings of the Royal Society of London. A. Mathematical and Physical Sciences 351(1666):351–380. 10.1098/rspa.1976.0146

[CR16] Fung YC (2013) Biomechanics. Mechanical Properties of Living Tissues; Springer Science & Business Media, New York. 10.1007/978-1-4757-2257-4 (**978-0-387-97947-2**)

[CR17] García-Vilana S, Sánchez-Molina D, Velázquez-Ameijide J, Llumà J (2021) Injury metrics for assessing the risk of acute subdural hematoma in traumatic events. Int J Environ Res Public Health 18(24):13296. 10.3390/ijerph182413296 34948905 PMC8702226

[CR18] García-Vilana S, Sánchez-Molina D, Llumà J, Galtés I, Velázquez-Ameijide J, Rebollo-Soria MC, Arregui-Dalmases C (2021) Viscoelastic characterization of parasagittal bridging veins and implications for traumatic brain injury. Bioengineering 8(10):145. 10.3390/bioengineering8100145 34677218 PMC8533420

[CR19] García-Vilana S, Sánchez-Molina D (2025) Statistical mechanics of bone damage: a constitutive model. Eur Biophys J 54(3–4):185–200. 10.1007/s00249-025-01749-9 40317307 PMC12106518

[CR20] García-Vilana S, Sánchez-Molina D (2022) Age effects on the mechanical behavior of human cerebral bridging veins. Clin Biomech 100:105792. 10.1016/j.clinbiomech.2022.105792 36327547

[CR21] García-Vilana S, Sánchez-Molina D (2024) Constitutive relationships for osteonal microcracking in human cortical bone using statistical mechanics. Continuum Mech Thermodyn 1–19. 10.1007/s00161-023-01257-1

[CR22] Gomes MS, Carmo GP, Ptak M, Fernandes FAO, Alves de Sousa RJ (2024) Accuracy and efficiency of finite element head models: the role of finite element formulation and material laws. Int J Numer Methods Biomed Eng 40(9):e3851. 10.1002/cnm.3851 39045773

[CR23] Guth E (1966) Statistical mechanics of polymers. Journal of Polymer Science Part C: Polymer Symposia 12(1):89–109. 10.1002/polc.5070120109

[CR24] Haider MA, Pearce KJ, Chesler NC, Hill NA, Olufsen MS (2024) Application and reduction of a nonlinear hyperelastic wall model capturing ex vivo relationships between fluid pressure, area, and wall thickness in normal and hypertensive murine left pulmonary arteries. Int J Numer Methods Biomed Eng 40(3):e3798. 10.1002/cnm.3798 38214099

[CR25] Ho J, Kleiven S (2007) Dynamic response of the brain with vasculature: a three-dimensional computational study. J Biomech 40:3006–3012. 10.1016/j.jbiomech.2007.02.011 17433331

[CR26] Holzapfel GA, Gasser TC, Ogden RW (2000) A new constitutive framework for arterial wall mechanics and a comparative study of material models. J Elast Phys Sci Solids 61:1–48. 10.1023/A:1010835316564

[CR27] Imanian A, Modarres M (2018) A thermodynamic entropy-based damage assessment with applications to prognostics and health management. Struct Health Monit 17(2):240–254. 10.1177/1475921716689561

[CR28] Joule JP (1859) On some thermo-dynamic properties of solids. Philos Trans R Soc Lond 149:91–131. 10.1098/rstl.1859.0005

[CR29] Khajehsaeid H, Tehrani M, Alaghehband N (2021) Anisotropic damage of soft tissues in supra-physiological deformations. J Biomech 124:110548. 10.1016/j.jbiomech.2021.110548 34171681

[CR30] Klika V, Pérez MA, García-Aznar JM, Maršík F, Doblaré M (2014) A coupled mechano-biochemical model for bone adaptation. J Math Biol 69(6):1383–1429. 10.1007/s00285-013-0736-9 24212399

[CR31] Kloczkowski A, Mark JE, Erman B, Eichinger BE (1991) Comment on: statistical mechanics of rubber elasticity. J Chem Phys 95(9):7015–7016. 10.1063/1.461049

[CR32] Kroon M, Holzapfel GA (2008) A new constitutive model for multi-layered collagenous tissues. J Biomech 41:2766–2771. 10.1016/j.jbiomech.2008.05.033 18657813

[CR33] Lee MC, Haut RC (1989) Insensitivity of tensile failure properties of human bridging veins to strain rate: implications in biomechanics of subdural hematoma. J Biomech 22(6–7):537–542. 10.1016/0021-9290(89)90005-5 2808439

[CR34] Lemaitre J (1985) Coupled elasto-plasticity and damage constitutive equations. Comput Methods Appl Mech Eng 51(1–3):31–49. 10.1016/0045-7825(85)90026-X

[CR35] Li W (2016) Damage models for soft tissues: a survey. J Med Biol Eng 36(3):285–307. 10.1007/s40846-016-0132-1 27441033 PMC4935756

[CR36] Li W (2016) ’damage models for soft tissues: a survey. J Med Biol Eng 36(3):285–307. 10.1007/s40846-016-0132-1 27441033 PMC4935756

[CR37] Migueis GFJ, Fernandes FAO, Ptak M, Ratajczak M, de Sousa RA (2019) Detection of bridging veins rupture and subdural haematoma onset using a finite element head model. Clin Biomech 63:104–111. 10.1016/j.clinbiomech.2019.02.010 30851565

[CR38] Miller JD, Nader R (2014) Acute subdural hematoma from bridging vein rupture: a potential mechanism for growth. J Neurosurg 120(6):1378–1384. 10.3171/2013.10.JNS13272 24313607

[CR39] Monea AG, Baeck K, Verbeken E, Verpoest I, Vander Sloten J, Goffin J, Depreitere B (2014) The biomechanical behaviour of the bridging vein–superior sagittal sinus complex with implications for the mechanopathology of acute subdural haematoma. J Mech Behav Biomed Mater 32:155–165. 10.1016/j.jmbbm.2013.12.007 24463477

[CR40] Monson KL, Converse MI, Manley GT (2019) Cerebral blood vessel damage in traumatic brain injury. Clin Biomech 64:98–113. 10.1016/j.clinbiomech.2018.02.011 29478776

[CR41] Natali AN, Carniel EL, Gregersen H (2009) Biomechanical behaviour of oesophageal tissues: material and structural configuration, experimental data and constitutive analysis. Med Eng Phys 31(9):1056–1062. 10.1016/j.medengphy.2009.07.003 19651531

[CR42] Neal-Sturgess CE (2002) A thermomechanical theory of impact trauma. Proc Inst Mech Eng Part D J Autom Eng 216(11):883–895. 10.1243/0954407023210314

[CR43] Nolan DR, Gower AL, Destrade M, Ogden RW, McGarry JP (2014) A robust anisotropic hyperelastic formulation for the modelling of soft tissue. J Mech Behav Biomed Mater 39:48–60. 10.1016/j.jmbbm.2014.06.016 25104546

[CR44] Popović M, Minceva M (2020) Thermodynamic properties of human tissues. Therm Sci 24(6B):4115–4133. 10.2298/TSCI200109151P

[CR45] Pukaluk A, Wolinski H, Viertler C, Regitnig P, Holzapfel GA, Sommer G (2023) Changes in the microstructure of the human aortic adventitia under biaxial loading investigated by multi-photon microscopy. Acta Biomater 161:154–169. 10.1016/j.actbio.2023.02.027 36812954

[CR46] Ramsey NF (1956) Thermodynamics and statistical mechanics at negative absolute temperatures. Phys Rev 103(1):20. 10.1103/PhysRev.103.20

[CR47] Rausch MK, Karniadakis GE, Humphrey JD (2017) Modeling soft tissue damage and failure using a combined particle/continuum approach. Biomech Model Mechanobiol 16:249–261. 10.1007/s10237-016-0814-1 27538848 PMC5288267

[CR48] Roy CS (1881) The elastic properties of the arterial wall. J Physiol 3(2):125–59. 10.1113/jphysiol.1881.sp000088 16991309 PMC1484813

[CR49] Sáez P, Alastrué V, Peña E, Doblaré M, Martínez MA (2012) Anisotropic microsphere-based approach to damage in soft fibered tissue. Biomech Model Mechanobiol 11:595–608. 10.1007/s10237-011-0336-9 21769621

[CR50] Sanchez-Molina D, Velazquez-Ameijide J, Arregui-Dalmases C, Rodríguez D, Quintana V, Shafieian M, Crandall JR (2014) A microcontinuum model for mechanical properties of esophageal tissue: experimental methodology and constitutive analysis. Ann Biomed Eng 42:62–72. 10.1007/s10439-013-0897-0 23975385

[CR51] Sánchez-Molina D, García-Vilana S (2024) Acoustic emission applied to stochastic modeling of microdamage in compact bone. Biomech Model Mechanobiol 23:1277–1287. 10.1007/s10237-024-01838-2 38553591 PMC11584445

[CR52] Sánchez-Molina D, Martínez-González E, Velázquez-Ameijide J, Llumà J, Soria MR, Arregui-Dalmases C (2015) A stochastic model for soft tissue failure using acoustic emission data. J Mech Behav Biomed Mater 51:328–336. 10.1016/j.jmbbm.2015.07.002 26282075

[CR53] Sánchez-Molina D, García-Vilana S, Llumà J, Galtés I, Velázquez-Ameijide J, Rebollo-Soria MC, Arregui-Dalmases C (2021) Mechanical behavior of blood vessels: elastic and viscoelastic contributions. Biology 10(9):831. 10.3390/biology10090831 34571709 PMC8472519

[CR54] Shenker O (2017) Foundation of statistical mechanics: mechanics by itself. Philos Compass 12(12):e12465. 10.1111/phc3.12465

[CR55] Swendsen RH (2017) Thermodynamics, statistical mechanics and entropy. Entropy 19(11):603. 10.3390/e19110603

[CR56] Toussaint R, Pride SR (2002) Fracture of disordered solids in compression as a critical phenomenon. I. Statistical mechanics formalism. Phys Rev E 66(3):036135. 10.1103/PhysRevE.66.036135 12366212

[CR57] Treloar LRG (1976) The Physics of Rubber Elasticity, 3rd edn. Clarendon Press, Oxford, p 1976. 10.1002/pi.4980080107

[CR58] Velázquez-Ameijide J, García-Vilana S, Sánchez-Molina D, Martínez-González E, Llumà J, Rebollo-Soria MC, Arregui-Dalmases C (2021) Influence of anthopometric variables on the mechanical properties of human rib cortical bone. Biomed Phys Eng Express 7(3):035013. 10.1088/2057-1976/abf787 33848994

[CR59] Vito RP, Dixon SA (2003) Blood vessel constitutive models-1995–2002. Annu Rev Biomed Eng 5(1):413–439. 10.1146/annurev.bioeng.5.011303.120719 12730083

[CR60] Wallace D (2020) The necessity of Gibbsian statistical mechanics. Determinism, indeterminism and laws of nature, In Statistical mechanics and scientific explanation, pp 583–616

[CR61] Wituła R, Hetmaniok E, Słota D (2012) A stronger version of the second mean value theorem for integrals. Comput Math Appl 64(6):1612–1615. 10.1016/j.camwa.2012.01.008

[CR62] Yang J, Zhao J, Liao D, Gregersen H (2006) Biomechanical properties of the layered oesophagus and its remodelling in experimental type-1 diabetes. J Biomech 39:894–904. 10.1016/j.jbiomech.2005.01.022 16488228

[CR63] Ye Y, Lan T, Zeng X, Yang J, Wei R, Zhu J, Liu M, Zhu X (2024) Bridging veins: an analysis of surgical anatomy and histology correlated with interhemispheric approaches. Front Neuroanat 18:1406252. 10.3389/fnana.2024.1406252 39669297 PMC11634616

